# Doubling down with CAR-T cell cancer immunotherapy: a two-step recognition circuit enables discrimination between target antigen high and low cancer cells

**DOI:** 10.1038/s41392-021-00706-0

**Published:** 2021-07-28

**Authors:** Jehad Charo, Bruno Gomes

**Affiliations:** 1 F. Hoffmann-La Roche AG, Pharmaceutical Research and Early Development Oncology, Roche Innovation Center Zurich, Zurich, Switzerland; 2 F. Hoffmann-La Roche AG, Pharmaceutical Research and Early Development Oncology, Roche Innovation Center Basel, Basel, Switzerland

**Keywords:** Tumour immunology, Tumour immunology, Preclinical research, Translational immunology

In a recent article published in Science by Hernandez-Lopez et al. chimeric antigen receptor (CAR) expressing T (CAR-T) cells with a two-step recognition system successfully discriminated between target cells expressing high and low levels of their target antigen, which enabled the specific rejection of solid tumour xenograft.^1^

CAR-T cells cancer therapies are approved as a treatment for B-cell lymphoma. A by-stander depletion of normal B cells—which express the same CD19 antigen targeted by the approved CAR-T therapies—is an acceptable risk in these haematological diseases. The progress of CAR-T cell immunotherapies in solid tumours has been lagging behind due to multiple factors including the tumour heterogeneity and immunosuppressive microenvironment and the lack of unique tumour targets that can be recognized by the CAR-T cells while sparing the healthy tissues. Tissue-specific overexpressed antigens such as HER-2 are attractive tumour targets because of their prevalence across patients and indications, and previous validation by other therapeutic modalities.^2^ However, HER-2-specific CAR-T cell immunotherapy has been hampered by the development of pulmonary toxicity in the first patient treated with the second generation of HER-2 targeting CAR-T cells. This toxicity was speculated to be triggered by the low level of HER-2 expressed on the lung epithelial cells.^3^ CAR-T cell immunotherapy can be genetically engineered to minimize the deleterious damages of on-target / off-tumour toxicities (or bystander healthy tissue damages) to mitigate life-threatening toxicities and achieve successful development in solid tumours.

**Fig. 1:**
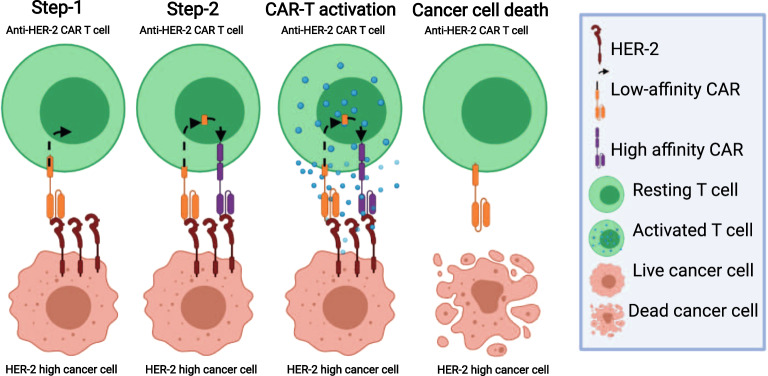
CAR-T cells utilize 2-steps recognition mechanism to specifically recognize and then kill cancer cells expressing high levels of HER-2. In step 1, HER-2 (in brown) high cancer cell is recognized by the low affinity CAR (in orange) on the CAR-T cells, which leads to the cleavage of fused transcription factor shown in step 2. This transcription factor initiate the transcription and expression of high affinity CAR (in purple). HER-2 high cancer cell is recognized by the high affinity CAR, which activates the CAR-T cell, ultimately leading to the killing of the HER-2 high cancer cell. Created with BioRender.com

Hernandez-Lopez et al. in a recent publication offered an elegant, new approach termed “two-step recognition circuit”.^1^ The authors have developed a synNotch-controlled gene expression system in which low affinity HER-2 specific CAR recognizing high, but not low, levels of HER-2 expressed on tumour cells induces the expression of a high affinity HER-2-specific 2nd generation CAR on the T-cell surface (Fig. 1). After confirming that this system is functional in recognizing tumour cells expressing high, but not low HER-2, they have used pairs of xenograft mouse models with high and low HER-2-expressing tumours injected contralaterally in order to demonstrate specific rejection of HER-2-high tumours upon the transfer of CAR-T cells expressing this circuit.

A key point to support further use and development of such an approach is the density discrimination of low vs. high antigen-expressing cells once CAR-T cells have been activated by high-density tumour cells, in order to spare low-density normal cells. Interestingly, the authors have shown that CAR-T cells with the two-step recognition circuit efficiently killed spheroids of HER-2-high tumour cells, kept some killing activity on proximal HER-2-low spheroids but lost this killing activity on more distant HER-2-low cells.^1^ This characteristic is beneficial as it enables killing of tumour variant with lower target antigen expression in the tumour microenvironment, while sparing more distal cells with low levels of the same target antigen. Hernandez-Lopez et al. utilized the sequence of 4D5-8 or trastuzumab, which is an established therapy in breast and gastric cancers. Trastuzumab specifically recognizes human HER-2, but not its mouse orthologue ERBB2, preventing the evaluation of whether the two-step recognition circuit can spare normal cells expressing physiological levels of ERBB2 in mouse models. Such an evaluation can be done using ERBB2 cross-reactive CAR or in a carefully planned clinical study.

Hernandez-Lopez et al. have also demonstrated that these CAR-T cells accumulate preferentially - but not exclusively - in tumours with high HER-2 expression. The authors did not comment on the dynamic of this accumulation or whether these cells were of activated phenotype, both of which could inform on potential long-term safety implication.^2^ The two-step recognition system can simply utilizes the extreme low and high affinity CARs reproducibly, as confirmed in vitro with a second target antigen (EGFR), obviating the need to evaluate and identify optimal CAR affinity for each new target.^1^

The temporal nature and dynamic range of the target gene expression on tumour compared to normal tissues can limit the success of synNotch-CAR-T system when targeting the same antigen. Interestingly, synNotch-CAR-T system can be used for building CAR-T cells with dual target recognition capacity that can further refine the specificity of CAR-T cells. This was explored in two subsequent articles. These articles demonstrated that synNotch-CAR-T can reject xenograft with heterogeneous expression of the tumour-specific target antigen epidermal growth factor receptor splice variant III (EGFRvIII) or alkaline phosphatase placental-like 2 (ALPPL2) when combined with targeting tumour-associated overexpressed or tissue-specific antigens, including ephrin type A receptor 2 (EphA2), interleukin 13 receptor α2 (IL13Rα2), melanoma cell adhesion molecule (MCAM), mesothelin, HER-2 or myelin oligodendrocyte glycoprotein (MOG).^4,5^ Interestingly, these studies revealed a significant added benefit of using the synNotch-CAR-T as compared to the constitutive CAR-T system, which is the increased persistence of synNotch-CAR-T. This was attributed, at least in part, to the preferential representation of stem cell memory–like T cells expressing the transcription factor T cell factor 1 (TCF1) and lower expression of the exhaustion marker CD39, potentially due to the temporary expression of the high affinity CAR.^4,5^

Additional questions remain to be explored for validating this and other CAR-T cells as a treatment for solid tumours. However, the two-step recognition circuit system is an elegant addition to the cancer immunology toolbox that could be further exploited to drive a more vigorous immune response by inducing a certain effector molecule or cytokine expression in addition to antigen specific CAR that can increase both the activity and durability of this therapy.
